# Progress in Diseases of the Nervous System

**Published:** 1904-03-26

**Authors:** 


### Progress in Diseases of the Nervous System.

**The Diagnostic Significance of Certain Reflexes.**—Oppenheim[1] of Berlin, in the course of a discussion on this question, said that the knee-jerk may, in very rare cases be congenitally absent, in association with other hereditary stigmata, or in children of syphilitic parents. It should also be remembered that mechanical conditions may render the demonstration of this reflex difficult or impossible, *e.g.* obesity, diseases of the knee-joint. The Achilles-tendon phenomenon—*i.e.*, plantar flexion of the foot on percussion of the tendo Achillis—is a much more constant phenomenon than is generally thought, especially if the patient be in a kneeling position on a chair. Sciatica causes a disappearance of the jerk, and in some cases the jerk never returns. Again, talipes valgus and arthritis of the ankle joint may act in a similar manner. The value of this reflex in establishing a diagnosis of tabes dorsalis is very great, provided the above conditions can be excluded. It should also be remembered that alcoholism and diabetes may also cause its disappearance. Babinski's phenomenon—dorsal flexion of the great toe on stroking the foot—nearly always indicates degeneration of the lateral pyramidal tracts. This degeneration also brings about hypertonicity of the muscles; but if there be an associated degeneration of the posterior tracts the latter may more than counterbalance the former, so that the muscles are hypotonic, and Babinski's sign be the only symptom of the pyramidal lesion. It appears that coincidental lesions of certain cerebral tracts may prevent the appearance of Babinski's phenomenon, though the pyramidal tract be degenerated.

**Tendon Reflexes in Uræmia.**—Stevens[2] says that in uræmia there is very commonly found an increase of muscular irritability with exaggeration of all tendon reflexes. This may be of distinct service in the diagnosis of the condition. Thus uræmic coma may simulate cerebral hæmorrhage, for the latter may be unaccompanied by paralysis and conversely uræmic coma may present hemiplegic symptoms of the nature either of convulsions or paralysis. Again both conditions are frequent in cases of granular kidney, with its characteristic effects on the pulse and heart. Occasionally it has been found that in cerebral hæmorrhage the knee-jerk on the paralysed side is exaggerated from the first, but in uræmia they are exaggerated on both sides. Again, uræmia may produce symptoms such as vomiting, headache, malaise, dyspnœa, sleeplessness, which do not attract attention to the state of the urine. In such cases an exaggerated reflex irritability may lead one to suspect that uræmia is impending.

**General Paralysis of the Insane.**—Robertson[3] says that the general opinion, "No syphilis, no general paralysis," is marred by the failure to take into account the facts, that only a very small proportion of syphilitics become general paralytics or tabetics, that syphilis is certainly absent in some cases, and that certain other conditions such as chronic alcoholism, excessive meat eating and lead poisoning, favour the development of general paralysis. He thinks that the disease is the result of a chronic toxic infection from the respiratory and alimentary tracts dependent on the excessive development of various bacterial forms, and especially on the abundant growth of diphtheroid bacillus. The evidence in support of this view is as follows: 1. The bone-marrow presents chronic changes of a severe character, and the bones are commonly thickened and sclerosed. 2. Various clinical facts—especially the rises of temperature, the leucocytosis, the disorders of the digestive functions, and the constantly existing chronic sore throat and septic condition of the mouth. 3. The chronic catarrhal condition of the alimentary canal, manifested by proliferative, atrophic, and sclerotic changes. 4. Direct evidence of general excessive development of saprophytic bacteria. 5. The almost constant presence in the respiratory and alimentary tracts of a bacillus which resembles the Klebs-Loeffler bacillus. 6. There is evidence that this diphtheroid bacillus occasionally takes part in a terminal blood invasion in cases of general paralysis. 7. A filamentous organism, in its individual segments indistinguishable from some forms of the diphtheroid organism, may be, with considerable frequency, observed in the lymphatics of the alimentary tract. 8. Experimental evidence shows that, when introduced by way of the alimentary tract in the form of broth cultures, the diphtheroid bacillus produces in the rat changes in the central nervous system having a distinct resemblance to those that occur in general paralysis.

**Histology of Nerve Cells.**—We owe a useful summary of recent researches on this subject to Williamson.[4] By Golgi's method of silver staining, the nerve cell and all its processes are made black. In the adult all nerve cells have processes, only in the embryo are nerve cells found without processes. Unipolar cells are only found in the posterior root ganglia, bipolar cells almost exclusively in the peripheral sensory nervous system. The common cells in the central nervous system are multipolar, one process (the axon) does not divide but passes into the axis cylinder of a nerve fibre, whilst the others—called dendrites—branch and subdivide. The internal structure of nerve-cells is shown by Nissl's staining method. The cells may be divided into (1) somatochrome cells—in which both nucleus and granules in the protoplasm are stained, and (2) carychrome cells—in which the nucleus only is stained. The anterior horn cells are typical somatochrome cells, the posterior horn cells and those of the gelatinous substance of Rolando are carychrome cells. The stained granules—chromophile or tigroid substance—are found in the cell substance and in the dendrites, not in the axis-cylinder. The unstained substance of the nerve cells is probably composed of fibrillæ, which give the appearance of a network. The nucleus of a nerve cell is globular, surrounded by a distinct membrane and has a large nucleolus. After section of a motor nerve, the nerve fibres undergo degeneration on the peripheral side, whilst the cells from which the axis cylinder arose also pass through certain changes. There is, first, the stage of solution of the chromophile substance. The granules become gradually dissolved, so that the cells stain uniformly, and are slightly enlarged, and the nuclei pass to the periphery of the cells. These changes may be seen in 40 hours, and last about a fortnight or three weeks. Some of the cells atrophy. Secondly, reparation occurs, in those cells which do not atrophy the granules reappear, the cell returns to is former size and the nucleus returns to its central position. In man the course of these changes is not quite the same as in animals. The stage of solution is not followed by one of repair, but goes on in all the cells to one of atrophy. After section of a peripheral sensory nerve chromatolysis, usually followed by recovery, takes place in the cells of the cells in the posterior root ganglion. But when the posterior nerve root is divided proximally to the ganglion no cell changes in the ganglion can be detected. And in man disease of the nerve fibres in the posterior columns does not bring about changes in the cells of the posterior root ganglia. Division of the pyramidal fibres is followed by chromatolysis of the large pyramidal cells in the motor cortex of the brain, and the cells completely atrophy. The same result occurs in man, *e.g.* after a hæmorrhage into the internal capsule. Section of the lateral cerebellar tract is followed by chromatolysis in the cells of the column of Clarke. Section of sensory spinal nerve roots is followed by chromatolysis of the posterior lateral group of nerve cells in the anterior horn. Primary changes are found in nerve cells in various pathological conditions—these vary considerably, they are due to various poisons, *e.g.* phosphorus, arsenic, alcohol, strychnine, uræmia, diphtheria. In acute anterior polomyelitis the nerve cell lesions appear to be dependent on vascular changes and the exudation of round cells. The same relation also occurs in many cases of acute myelitis and in syphilitic cord diseases associated with vascular changes.

[1] Med. Rec., Jan. 2. [2] Brit. Med. Jour., Jan. 16. [3] Ibid. Oct. 24. [4] Med. Chron. Oct.

